# Clay mineralogy indicates a mildly warm and humid living environment for the Miocene hominoid from the Zhaotong Basin, Yunnan, China

**DOI:** 10.1038/srep20012

**Published:** 2016-02-01

**Authors:** Chunxia Zhang, Zhengtang Guo, Chenglong Deng, Xueping Ji, Haibin Wu, Greig A. Paterson, Lin Chang, Qin Li, Bailing Wu, Rixiang Zhu

**Affiliations:** 1 Key Laboratory of Cenozoic Geology and Environment, Institute of Geology and Geophysics, Chinese Academy of Sciences, Beijing 100029, China; 2 CAS Center for Excellence in Tibetan Plateau Earth Sciences Beijing 100101, China; 3 State Key Laboratory of Lithospheric Evolution, Institute of Geology and Geophysics, Chinese Academy of Sciences, Beijing 100029, China; 4 Department of Paleoanthropology, Yunnan Institute of Cultural Relics and Archeology, Kunming 650118, China; 5 Institute of Vertebrate Paleontology and Paleoanthropology, Chinese Academy of Sciences, Beijing 100044, China; 6 Key Laboratory of Earth and Planetary Physics, Institute of Geology and Geophysics, Chinese Academy of Sciences, Beijing 100029, China

## Abstract

Global and regional environmental changes have influenced the evolutionary processes of hominoid primates, particularly during the Miocene. Recently, a new *Lufengpithecus cf. lufengensis* hominoid fossil with a late Miocene age of ~6.2 Ma was discovered in the Shuitangba (STB) section of the Zhaotong Basin in Yunnan on the southeast margin of the Tibetan Plateau. To understand the relationship between paleoclimate and hominoid evolution, we have studied sedimentary, clay mineralogy and geochemical proxies for the late Miocene STB section (~16 m thick; ca. 6.7–6.0 Ma). Our results show that *Lufengpithecus cf. lufengensis* lived in a mildly warm and humid climate in a lacustrine or swamp environment. Comparing mid to late Miocene records from hominoid sites in Yunnan, Siwalik in Pakistan, and tropical Africa we find that ecological shifts from forest to grassland in Siwalik are much later than in tropical Africa, consistent with the disappearance of hominoid fossils. However, no significant vegetation changes are found in Yunnan during the late Miocene, which we suggest is the result of uplift of the Tibetan plateau combined with the Asian monsoon geographically and climatically isolating these regions. The resultant warm and humid conditions in southeastern China offered an important refuge for Miocene hominoids.

The mid to late Miocene is an important period for the evolution of hominoid primates (apes and humans). Hominoid species flourished in East Africa and Europe around the mid Miocene Climate Optimum (17–15 Ma)^1^,^2^. Globally, however, the number of hominoid remains decreased sharply during the late Miocene with fossil remains from only Africa and parts of Asia. The southeast margin of the Tibetan Plateau, in particular, appears to have been an important habitat for late Miocene hominoids^3^,^4^,^5^,^6^. Several late Miocene hominoid fossils (Fig. 1a) have been found in Yunnan Province, southwestern China^4^,^5^. These hominoid fossils were all attributed to the genus *Lufengpithecus* and have been assigned to three species (summarized and in ref. 5): *L. keiyuanensis* from Kaiyuan in the Xiaolongtan Basin (~11.6–12.5 Ma)^7^,^8^, *L. hudienensis* from multiple sites in the Yuanmou Basin (7.1 ~ 9.0 Ma)^9^,^10^,^11^, *L. lufengensis* from Shihuiba in the Lufeng Basin (6.2–6.9 Ma)^12^ and *L. cf. lufengensis* from Shuitangba (STB, Fig. 1b) in the Zhaotong Basin (6.2 Ma)^5^,^13^. Therefore, the southeastern margin of the Tibetan Plateau, particularly in Yunnan, southwest China, appears to have served as an important refuge for the late Miocene hominoids^4^.

Paleoenvironmental reconstructions for the period of hominoid living provide important clues to understand environmental changes and the evolution of early hominoids in eastern Asia. Previous studies have reconstructed the paleoenvironments for Yunnan hominoids using pollen data, clay minerals, or fauna (Table 1). Both *L. keiyuanensis* in the Xiaolongtan Basin^14^ and *L. hudienensis* in the Yuanmou Basin^15^ were present in a warm and wet forested habitat. A predominantly forested habitat, with marginal environments consisting of bush-grassland^11^, subtropical, hilly, with some dense forestry and partial transitional bushes and seasonal climate prevailed for *L. hudienensis* in the Yuanmou Basin^9^,^16^. *L. lufengensis* in the Lufeng Basin was present in a moist southern subtropical-tropical environment when climate changed from warm and humid to warm and drier before becoming cooler, but still humid^17^,^18^. A transitional area of mixed forest and grassland under a warm and humid climate prevailed for *L. cf. lufengensis* in the Zhaotong Basin^19^. This diverse range of habitats and climates illustrates that the relationship between environmental changes and hominoid evolution in Yunnan is complex and remains ambiguous. Due to the limited availability of high-resolution palaeoenvironmental data and systematic comparisons for these critical areas, few studies have discussed the reasons for the existence of the refuge in the southeastern margin of the Tibetan Plateau.

The discovery of genus *Lufengpithecus* in the STB section of the Zhaotong Basin, northeastern Yunnan was magnetobiostratigraphically dated at ~6.2 Ma and thus serves as the youngest Miocene ape fossil^5^. The terminal Miocene was a critical time interval for the evolution of apes and early humans because this is when the divergence of ape and human lineages occurred in Africa^20^ and apes disappeared in Europe, later becoming extinct in East Asia^4^,^21^. Moreover, environmental changes as well as tectonic processes during this period, such as an expansion of C_4_ plants around the world in the late Miocene and Pliocene^22^,^23^,^24^, may had a profound influence on habitats and thus may have had an important role in the evolution of hominoids. Therefore, the STB site provides an important opportunity to explore detailed information on the living environment of late Miocene hominoids.

In this study, the 16 m thick STB section, which is dated as ~6.73–6.03 Ma^5^,^13^, was selected for sedimentary (Fig. 1c), mineralogical, and geochemical analyses (Fig. 2) to acquire high-resolution information on the hominoid living environment. This is combined with a comprehensive comparison of mid to late Miocene records of hominoid sites from Yunnan, Siwalik in Pakistan, and tropical Africa to reveal the relationship between paleoenvironment and hominoids during the late Miocene in East Asia.

## Results

### Sedimentary lithology

**Figure 1:**
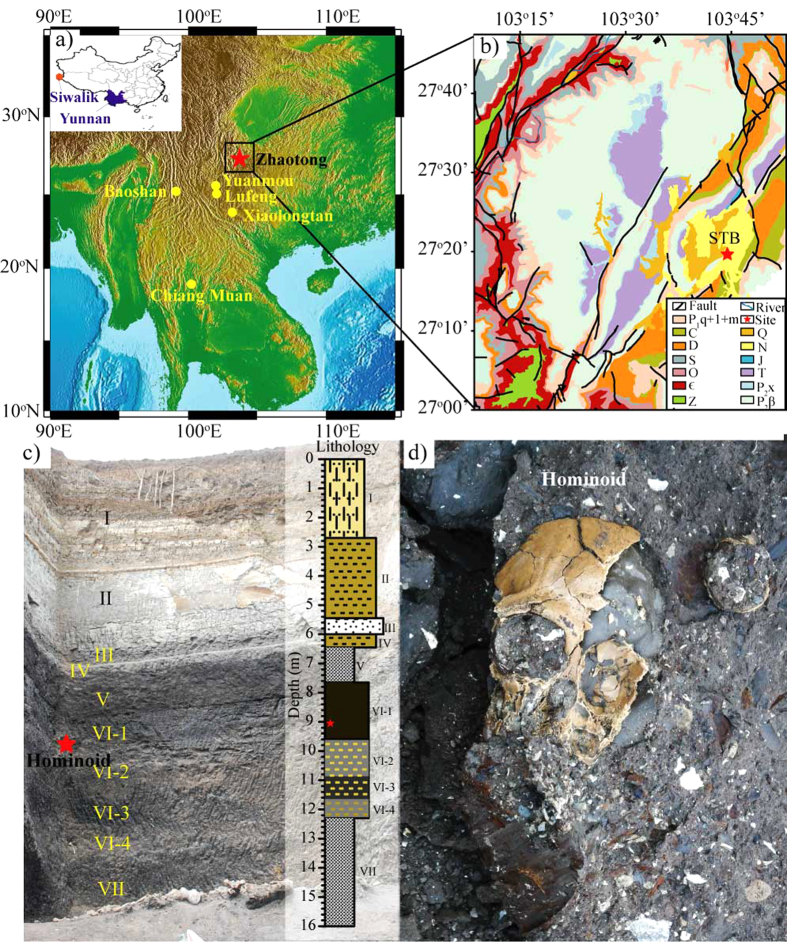
(**a**) The location of the study area (Zhaotong) and four other Miocene hominoid bearing sites in Yunnan Province, China; the map was made using GMT^58^. (**b**) Schematic geology map around the Shuitangba (STB) section in Zhaotong basin. (**c**) Lithological layer pictures and sketch of the 16 m thick STB section and the numbers represent the sedimentary layers. (**d**) A close up view of the black peaty layer to highlight the location of the *L. cf. lufengensis* cranium, which is shown and described in detail by Ji *et al.*, (2013)^5^. In part (**b**) the lithology symbols are as follows: Z: phyllite, quartz sandstone and carbonate; ϵ: dolomite, marlite and shellstone; O: shellstone, sandstone, dolomite and limestone; S: shellstone, sandstone, siltstone and limestone; D: sandstone, shellstone, dolomite and limestone; C: limestone, sandstone and dolomite; P_1_: limestone, shellstone, sandstone and dolomite; P_2_: Emeishan balsalt; T: sandstone, siltstone, marlite, dolomite; J: mudstone, siltstone, sandstone and marlite; K: sandstone; N: clay, lignite and sandy conglomerate; Q: gravel, sand and clay.

**Figure 2:**
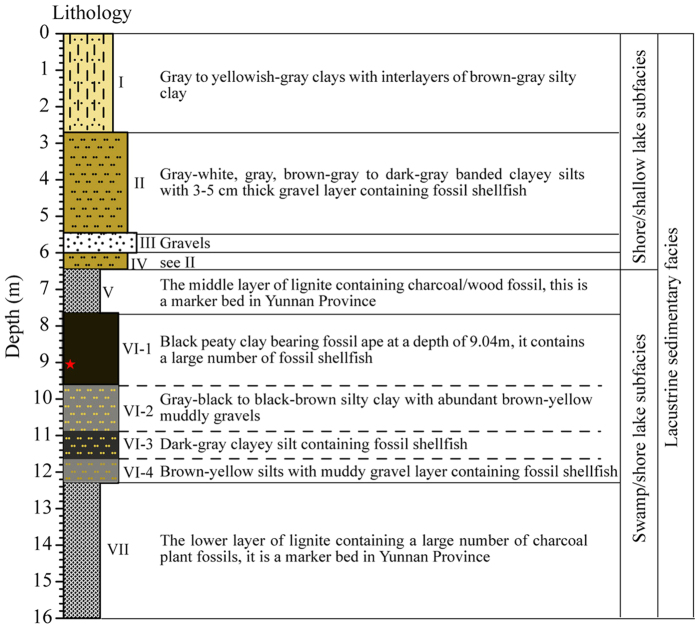
Lithologies and interpreted depositional facies from the STB section.

The lithology of the studied section is described in Fig. 2 (see Figs 1c and 2) and follows a typical lacustrine sedimentary facies sequence. Based on detailed sedimentary lithological information, sedimentary subfacies in the STB section can be recognized, bottom upwards, as follows: swamp subfacies (VII), shore lake subfacies (VI), swamp subfacies (V) and shore/shallow lake subfacies (IV-I).

### Clay mineralogy

**Figure 3:**
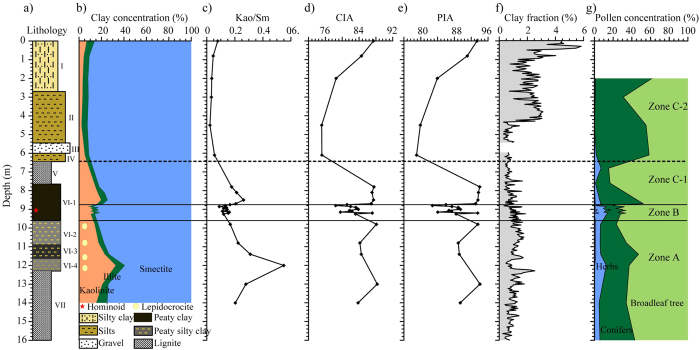
Stratigraphic variation of several proxies from the STB section. (**a**) Lithology. (**b**) Clay mineral composition (%). (**c**) The ratio of Kao/Sm (Kaolinite/Smectite). (**d**) CIA, and (**e**) PIA, chemical weathering indices for the isolated clay fractions (<2 μm). (**e**) Clay-fraction content. (**f**) Pollen concentrations from Chang *et al.* (2015)^19^. Shaded circles in (**b**) indicate the presence of lepidocrocite.

X-ray diffraction analyses on the clay fractions (<2 μm) from the whole section indicate that three main clay minerals dominate the STB section (smectite, kaolinite and illite), which are independent of lithology (Supplementary Fig. 1). Smectite is the dominant clay mineral with an average of 82.3%. Kaolinite is variable from 2% to 32.5% with an average of 12.3%. The concentration of illite is generally low, with an average of 5.2%. Lepidocrocite is present in some specimens, but only occurs in the depth interval of 12.0–9.8 m (Fig. 3b). The variations of the clay minerals assemblages and the ratio of kaolinite/smectite (Kao/Sm) with depth are shown in Fig. 3b,c. To explore the environmental changes associated with the appearance of *L. cf. lufengensis*, the section was divided into three zones from bottom to top: Zone A, B, and C (Fig. 3). Based on clay mineral concentrations, Zone C is further divided into subzones C-1 and C-2.

In Zone A (14.0–9.6 m, the period before *L. cf. lufengensis* appearance), the average concentrations of smectite, kaolinite and illite are 73.6%, 20.4% and 6.0%, respectively; the highest concentration of kaolinite is at the depth of 12 m (Fig. 3b). In Zone B (9.6–8.8 m, the period when *L. cf. lufengensis* was present), concentrations of clay minerals display smaller fluctuations and the ordered average values are smectite > kaolinite > illite (Fig. 3b). In Zone C (8.8–0 m, the period after *L. cf. lufengensis’s presence*), the average concentrations of smectite, kaolinite and illite in Zone C-1 are 78.0%, 16.7% and 5.3%, respectively. However, concentrations of clay minerals are more stable and the ordered average values in Zone C-2 are smectite > illite > kaolinite. The lowest concentration of kaolinite is at a depth of 4.5 m (Fig. 3b). The average values of Kao/Sm have the following order: Zone A > Zone C-1 > Zone B > Zone C-2 (Fig. 3c), which follows the same trend as kaolinite concentration (Fig. 3b).

### Geochemical analysis

We use the chemical index of alteration (CIA) and the plagioclase index of alteration (PIA) of the clay fractions, to indicate the degree of chemical weathering. As shown in Fig. 3d,e, a three-stage pattern is found in the studied sediments, which matches closely the clay minerals characteristics in Fig. 3b,c. The CIA and PIA values are in the range of 75–88, and 77–94, respectively. The variations of CIA, and PIA values with depth are consistent with each other and their average values have the same following order: Zone C-1 > Zone A > Zone B > Zone C-2 (Fig. 3d,e).

### Scanning Electron Microscope (SEM) analysis

**Figure 4:**
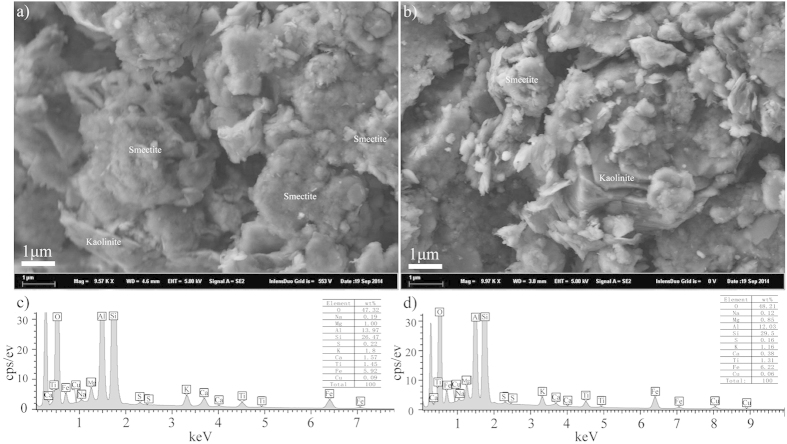
SEM images and EDX analysis results of clay-fraction (<2 μm) samples at depths of (a,c) 12 m and (b,d) 9.06 m in the STB section.

Clay fractions of samples from 12 m and 9.06 m were analyzed by SEM and energy spectrum analysis (EDX). As shown in Fig. 4, irregular fluffy masses of extremely small smectite particles are dominant, with some lager masses, which appear to be stacks of flake-shaped units without a regular outline (Fig. 4a). Well-crystallized kaolinite particles with well-formed six-sided flakes are found in Fig. 4b. These particles appear to be twinned and a few kaolinite particles with a prominent elongation in one direction also are found in Fig. 4a. EDX results (Fig. 4c,d) of the whole surfaces of clay fractions are almost homogeneous and both show dominant peaks of O, Si and Al, along with smaller peaks of Fe. Only small concentrations of other elements are observed.

## Discussion

Both the lithology and sedimentary facies of the sequence can potentially influence the mineralogy and geochemistry. However, the clay mineral species (smectite, kaolinite and illite; Fig. 3b) and clay-fraction content (Fig. 3f) are independent of the lithology (Fig. 3a) and sedimentary facies (Fig. 2) in the STB section. The assemblages of clay minerals in the STB section are smectite, kaolinite and illite, which are same clay mineral assemblage of topsoil derived from direct weathering of the Emeishan basalts near the STB site (the sample with the depth of 0 m in Fig. 3a). Permian limestone and Emeishan basalts are the main basement rocks of the Zhaotong Basin and the region in general (the main basements of catchment around the sampling site are also Emeishan basalts; Fig. 1b). Therefore, clay fractions in sampling site are mainly derived from the weathering of Emeishan basalts in this area. The formation of clay minerals in the STB section is likely controlled by the climate. Du *et al.* (2012)^25^ demonstrated that, as climate changes from dry and cold in north China to humid and hot in south China, the assemblages of clay minerals in the weathering products of basalts changes in the following fashion: smectite + illite + kaolinite → smectite + kaolinite → kaolinite + gibbsite. Smectite commonly develops abundantly in low reliefs where poor drainage prevents the removal of silica, alkaline and alkaline-earth ions. This is generally linked to a warm climate with alternating humid and dry seasons^26^. In contrast, kaolinite is generally a product of highly hydrolytic weathering reactions and forms in perennially warm humid climates with a minimum temperature of ~15 °C^26^. Therefore, the relative proportions of smectite and kaolinite can be used as a proxy for climate, whereby a high kaolinite/smectite (Kao/Sm) ratio is indicative of humid/warm to more dry and seasonal climate variations. Previous work has also demonstrated that the geochemical characteristics of clay fractions are more sensitive to climate than that of bulk rock, especially siltstone and sandstone^27^. Therefore, combined analysis of clay assemblages and geochemistry can be used for paleoclimate reconstructions.

Based on the sedimentology, clay mineral, and geochemical analyses of clay-fraction on the STB section, the palaeoclimate environments before, during and after the presence of *L. cf. lufengensis* are discussed.

In Zone A (16.0–9.6 m, the period before *L. cf. lufengensis* appeared), the lithology gradually changes from a lower layer of lignite (16.0–12.4 m, formation VII) to silt, clayey silt, and silty clay with a large number of fossil shellfish (12.4–9.6 m, formation VI-2, 3 and 4). Normally, lepidocrocite is formed in anoxic conditions^28^ and the presence of lepidocrocite in the sediments from the depth of 12.0–9.6 m (Fig. 3b) suggests an anoxic environment with relatively stagnant water during this period. This indicates that the sedimentary facies changed from a swampy lacustrine-marsh subfacies (coaly facies) to a shore lake subfacies with stagnant water, which is supported by dark color of these organic rich sediments. Only small quantities of clay were separated from the lignite layer at 16.0–12.4 m. This zone has the highest kaolinite content with well-crystallized particles (Fig. 4b). It also has the lowest smectite content (Fig. 3b) and the highest average value of Kao/Sm (Fig. 3c), which suggests a warm and humid environment. The CIA values of 84–88 (Fig. 3d) indicate strong chemical weathering^29^. The above mentioned observations suggest that, prior to the appearance of *L. cf. lufengensis*, the STB site experienced a change from a swampy lacustrine-marsh subfacies to a shore lake conditions under a warm and humid climate.

Within Zone B (9.6–8.8 m), which contains the *L. cf. lufengensis* fossils, the main sediment is black peaty clay (VI-1) that contains a large number of fossil shellfish, but with no lepidocrocite (Fig. 3c), which suggests a shallow water/swampy environment. Zone B has higher smectite content and lower kaolinite content than Zone A (Fig. 3b). Kao/Sm and CIA values vary in the range of 0.09–0.17 and 78–83, respectively (Fig. 3c,d). The average values of these two proxies are lower than those in Zone A, which indicates that the climate was mildly warm and humid when *L. cf. lufengensis* was present in this region.

Zone C (8.8–0 m, the period after *L. cf. lufengensis* appearance) is divided into two subzones, C-1 and C-2 (Fig. 3). In Zone C-1 (8.8–6.4 m), the sediments change from black peaty clay (the upper part of formation VI-1) into the second layer of lignite (formation V). This suggests that the environment was a swampy lacustrine-marsh subfacies (coaly facies) environment. Transitioning from Zone B to C-1, Kao/Sm and CIA values exhibit distinct increases (Fig. 3). This indicates a relatively sharp increase in temperature and humidity in this region. The sediments in Zone C-2 (6.4−0 m) including clayey silts (IV), gravel (III), clayey silts (II), silty clay and clay layers (I) (Figs 1c, 2), are recognized as shore/shallow lake subfacies (Fig. 2). The clay concentrations are characterized by dominant smectite (>90%) and low kaolinite (<8%) and are independent of lithological changes (Fig. 3a). Low Kao/Sm (Fig. 3c) and CIA values in the range of 75–87 (Fig. 3d) indicate that the climate became more seasonal/monsoonal during this period.

Changes in clay minerals and geochemical results are consistent with the pollen data (Fig. 3g) from Chang *et al.* (2015)^19^. Briefly, the environment recorded in the STB section before the appearance of *L. cf. lufengensis* (Zone A) was a relatively warm and humid environment that was mainly forested with subtropical to temperate evergreen and deciduous broad-leaved taxa. Mildly warm and humid conditions with a mixed sparse and miscellaneous wood forest types, including boreal vegetation prevailed when *L. cf. lufengensis* was present (Zone B). Following this, the climate rebounded to warm and humid conditions, which were followed by a gradual transition to cool and dry conditions with coniferous forests.

As discussed above, we draw the conclusion that, in the Zhaotong region, *L. cf. lufengensis* lived in mildly warm and humid conditions (Zone B) rather than the relatively warm and humid conditions that prevail prior to and following its appearance (i.e., Zones A and C-1). The lithological characteristics at the STB hominoid site are characterized by alternation between two layers of lignite and carbonaceous muds (Figs 1c–e and 2). Many charcoal plant fossils were found in the lignite, particularly in the lower lignite layer. This lignite consists of soft brown coal with low maturity and good bioactivity and the organic content is generally associated with higher plant content and lower grade aquatic organisms^30^. These plant materials were probably autochthonous in the lignite swamps. The alternation between carbonaceous muds and two layers of lignite (Figs 1c and 2) suggests sediment accumulation in standing or slow moving water of varying depth^31^. Vertebrate fossils occur only in layer VI, which consists of fine, dark and yellow-colored clays intercalated between layers of lignite. These vertebrate fauna have been characterized by Jablonski *et al.* (2014)^13^ who describe the environment as a densely vegetated, moist-forest paleoenvironment at the margin of standing water. Pollen analysis^19^ showed that the vegetation was dominated by subtropical evergreen broad-leaved taxa with a few temperate deciduous taxa (e.g., *Quercus*, *Castanea/Castanopsis*, *Alnus*) in the lignite layers and aquatic plants appeared in layer VI. The fossils of *Euryale*, an aquatic plant living in swamps and lakes with shallow and stable water, were also found in the hominoid-bearing layer^32^.The results of sedimentary facies, vertebrate fauna, pollen, and clay mineral analyses all indicate that *L. cf. lufengensis* was present in the Zhaotong region in lacustrine or swamp environments with mildly warm and humid climate conditions during the terminal Miocene.

Previous studies have indicated that hominoids became extinct throughout Eurasia during the late Miocene^33^. Five hominoid fossil sites have been found in Yunnan (Fig. 1a). A new mid-Miocene hominoid, *cf. Khoratpithecus chiangmuanensis* from northern Thailand was reported by Chaimanee *et al.* (2003, 2004)^6^,^34^. Therefore, the Yunnan and Thailand fossil apes provide a unique temporal perspective on the evolutionary history of hominoids in Southeast Asia. As shown in Table 1, they occurred during the mid to late Miocene (ca. 13.5−6.2 Ma) and are preserved in similar sedimentary facies (e.g., lignite, clay or alternative layers of lignite and peaty clay^31^). As summarized in Table 1, the hominoid living environments seem to have changed from warm and wet forested habitat to transitional environments with mixed forest and grassland in Yunnan and Thailand.

To compare environmental conditions when hominoids were present in Yunnan, biome reconstructions of three hominoid fossil sites were used (Supplementary Fig. 2). This approach is based on the biomization method developed by Member of China Quaternary Pollen Database (MCQPD)^35^ and improved by Wu *et al.*, (2014)^36^. The dominant vegetation in Xiaolongtan, Lufeng and Zhaotong sites are broadleaved evergreen/warm mixed forest (WAMF), temperate deciduous forest (TEDE), and transition between WAMF and TEDE, respectively. Fossil wood associated with the Yuanmou hominoid in Yuanmou Basin suggests that it was present in a subtropical broad-leaved forest environment. All of the above reconstructions indicate that the dominant vegetation was forest in Yunnan Province, at least during the period of ca. 12.5−6.2 Ma. On the other hand, the Miocene hominoid *Sivapithecus* is identified in several parts of the long Siwalik section between 12.7 and 6.8 Ma from the Potwar Plateau, Pakistan (part of the Indian subcontinent)^1^,^37^. Both fauna analyses^38^ and stable isotope analyses^22^ from the Pakistan Siwalik section suggest that at ~7 Ma a major faunal turnover occurred where vegetation transitioned from C_3_ to C_4_^22^,^38^,^39^. Hoorn *et al.*, (2000)^40^ examined vegetation changes in the Siwalik Group of Nepal in the Himalayan foothills and Gangetic floodplain, and, when combined with the vegetation reconstruction by Wu *et al.*, (2014)^36^, indicates that the dominate vegetation was TEDE between ca. 11.5−8 Ma, and WAMF between ca. 8−7 Ma, and steppe (STEP) after ca. 7 Ma. This indicates that a forested environment was replaced with grassland vegetation, which became well established after ca. 7 Ma. We conclude that the turnover of ecology in the Siwalik Group in the Himalayan foothills and floodplains was at ca. 7 Ma. In Yunnan Province however, the dominant vegetation was forest and this did not significantly change during the period of 12.5−6.2 Ma. Therefore, the vegetation changes in Siwalik and Yunnan were not synchronous.

**Figure 5:**
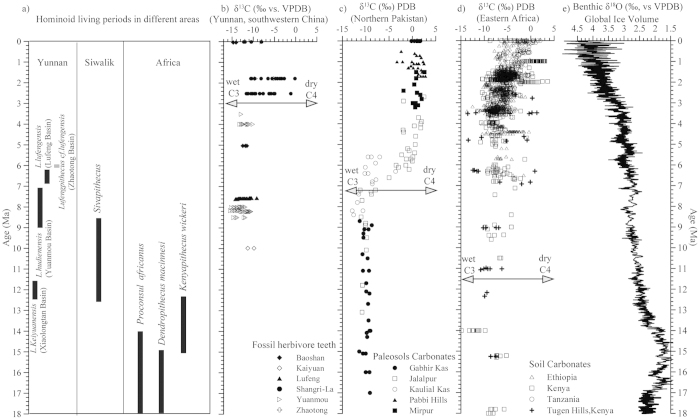
Comparison of hominoid sites in Asia and Africa. (**a**) Hominoid occurrences in Southeast Asia, Siwalik and Africa. Carbon isotope changes from (**b**) Yunnan^41^, (**c**) Siwalik in Northern Pakistan^39^, and (**d**) East Africa^42^. (**e**) Global ice volume as indicated by marine oxygen isotope^43^.

To obtain a better understanding of the relationship between paleoclimate and hominoid evolution in Asia, we summarize hominoid living periods from Yunnan, Thailand, Siwalik and Africa together (Fig. 5a) and compare Yunnan climate records (Fig. 5b^41^) with those from Nepal (Fig. 5d^39^), Africa (Fig. 5d^42^), and the marine oxygen isotope records (Fig. 5e^43^). The number of ape remains sharply decreased during the Late Miocene in Africa, particularly after ca. 12 Ma^2^,^44^. However, *Sivapithecus* remained in the Siwalik area during the period of ca. 12.7−6.8 Ma^45^ and ape remains are found in Southeast Asia from ca.13.5 Ma^34^ to at least 6.2 Ma^5^. The distribution of ape remains in these areas may be controlled by paleoclimate changes.

Temporal variations in the δ^13^C of bulk enamel samples from Yunnan Province (Fig. 5b) indicates that mammals on the southeast side of the Himalayan-Tibetan Plateau continued to feed primarily on C_3_ vegetation and lived in an environment dominated by dense forest until ~3–4 Ma^41^, which is consistent with limited pollen data from Dali basin in Yunnan^46^. Unlike the paleoclimate changes in Yunnan, stable carbon isotope data from paleosols in the Siwalik sediments (Fig. 5c) indicate that floodplain forests were replaced by C_4_ grasslands around 7−8 Ma^39^; 3–4 Myr earlier than in Yunnan. These stable carbon isotope changes in Yunnan and the Siwalik area are consistent with vegetation changes during the late Miocene (Supplementary Fig. 2). Unlike records from Yunnan and Siwalik, variations of stable carbon isotopes from soil carbonates in Africa do not reveal a clear shift that would be indicative of a distinct vegetation change (Fig. 5d). However, Bonnefille *et al.*, (2010)^47^ demonstrated that an expansion of savanna/grassland occurred at 10 Ma in East Africa and 7 Ma in West Africa and that arid conditions with scarce tree cover prevailed over tropical Africa between 6.3 and 6.0 Ma.

In summary, the ape remains in Yunnan, Siwalik and Africa are contemporaneous with the presence of forest vegetation in these areas (Fig. 5). Prior to ca. 7 Ma, African forests were progressively replaced by savanna/grassland, however, forests were still predominant in the Yunnan and Siwalik areas. Yunnan, at the southeast margin of the Tibetan Plateau, remained a warm and humid forested area until ca. 3–4 Ma, after which cold and dry conifer forest became dominant^46^. It is likely that these vegetation shifts were controlled by global and regional climate changes.

Global deep-sea δ^18^O isotope values (Fig. 5e) continued to rise steadily from the mid Miocene (~14 Ma) until the late Miocene (6 Ma), which indicates a gradual global cooling during this period^43^ that may have played a role in the aridification of Africa. The pivotal role of the shrinkage of the Tethys Sea during ~11−7 Ma for north African aridification was identified by Zhang *et al.*, (2014)^48^. The abrupt ecological shift in the Siwalik areas at ca. 7 Ma mainly resulted from climate changes related to the decreased elevation of the Himalayan foreland^36^. During the late Miocene, the dominant forest vegetation under warm and wet climate conditions in Yunnan was likely controlled by the Asian monsoon. The Asian monsoon was established during the late Oligocene to the early Miocene^49^ and reached maximum strength around 7–8 Ma^22^,^50^. The southwest monsoon system appeared in the late to middle Miocene, and intensified in the late Miocene when the modern southwest monsoon regime became permanently established^51^. In addition to the Asian Monsoon, the regional uplifts of the southeastern margin of the Tibetan Plateau^52^ have significantly affected the Xiaolongtan, Lufeng, Yuanmou and Zhaotong basins within or adjacent to the Xianshuihe-Xiaojiang fault system^53^. The survival of the hominoids in Yunnan may have benefited from episodes of uplift in the southeastern margin of the Tibetan Plateau, the impacts of which on regional climatic conditions may have been an important contributing factor in isolating the hominoids geographically and ecologically^4^.

Through a comprehensive comparison among records from Yunnan hominoid sites, Siwalik and tropical Africa during the late Miocene, we have found that humid to arid ecological shifts in tropical Africa and Siwalik are contemporaneous with the disappearance of hominoid fossil remains. Temporally, these vegetation shifts occur first in Africa (~7–10 Ma) and then Siwalik (~7 Ma), but similar changes are not observed in Yunnan until much later (~3–4 Ma) when vegetation changes from humid and warm to cold and dry forest types. This is consistent with the late Miocene *L. cf. lufengensis* fossil remains in the Zhaotong basin being present during a period of mildly warm and humid climate dominated by forest vegetation. It is likely that the uplift of the Tibetan Plateau combined with the Asia monsoon, geographically and climatically isolated the Yunnan region and delayed the onset of aridification. The warm and humid forests condition in Yunnan during late Miocene may have been an important contributing factor in isolating southern China (and presumably southeast Asia in general) and preserving environmental conditions favorable to hominoids in this area.

## Methods

### Geology, chronology and sampling

The Zhaotong Basin is located at the southeastern margin of the Tibetan Plateau with an elevation of 1900 to 2000 m, 380 km NNE of Kunming City, capital of Yunnan Province (Fig.1a). The Zhaotong Basin is one of the fault-related basins in the Xianshuihe-Xiaojiang fault zone that is a major fault zone in the South China fold belts^54^. A disconformable relationship is found between the stratigraphic base Permian limestone or Emeishan basalts and the overlying late Cenozoic sediments (Fig.1b). The basin is filled with late Miocene to Pliocene lacustrine or swampy clays, silts, peaty clays and lignites. This kind of peaty clay sediment is extensively distributed in late Neogene basins of the southeast margin of the Tibetan Plateau^55^,^56^. The age of Cenozoic sedimentary sequence in Zhaotong Basin is ~8.8−2.6 Ma^5^.

In this study we focus on the STB section (27° 19′ 41.8″N, 103° 44′ 13.7″E), which is in an open-pit lignite mine in the Zhaotong Basin. The thickness of the outcropping section is approximately 16 m. Detail magnetostratigraphic and biostratigraphic investigations of the STB section place stringent age control on the hominoid-bearing stratum. Ji *et al.*, (2013)^5^ and Jablonski *et al.*, (2014)^13^ suggested that the sequence spanned a time range of about 6.73−6.03 Ma, and the hominoid-bearing layer was estimated at around 6.2 Ma.

A total 337 samples with 5 cm intervals were collected from the STB section and 32 samples were selected from different parts in the section for analysis. Twelve samples were chosen from the hominoid-bearing layer (8.70−9.25 m), in order to obtain a higher resolution record during this time period.

### X-ray diffraction (XRD) analysis

Clay fractions were separated following the methods described in detail by Zhang and Guo (2013)^57^. Twenty-eight clay fraction samples were obtained from different layers, with the exception of the two layers of lignite, where insufficient clays were present. Clay mineralogy and element geochemistry were analyzed on carbonate-free particles. Clay mineralogy was analyzed by X-ray diffraction (XRD) using a PANAlytical diffractometer fitted with CuKα-radiation at 40 KV and 40 mA.

Clay minerals were identified on oriented slides of clay-sized (<2 μm) particles. The oriented slides were obtained following the methods described by Zhang and Guo (2013)^57^. Four XRD runs were performed after either air-drying, ethylene-glycol solvation, and after heating at 300 °C or 550 °C for 2 hours, respectively. Identification of clay minerals was performed mainly according to the position of the (001) series of basal reflection on the three XRD diagrams. Detailed descriptions of the identification procedures are given by Zhang and Guo (2013)^57^. Clay minerals smectite, illite and chlorite are recognized in all samples as shown in Supplementary Fig. 1. Magnetic mineral lepidocrocite was identified based on the peaks of 6.20 Å, which is evident on AD-slides and eg-slides, but not the 300 °C and 550 °C slides (e.g., Fig. 3d). Semi-quantitative estimates of peak area of the basal reflections for the main clay mineral groups of smectite (17 Å, eg-slide), illite (10 Å), and kaolinate (7 Å) were carried out on the glycolated curves using the MacDiff software with the weighting factors introduced by Biscaye (1965).

### X-Ray Fluorescence (XRF) analysis

Major elements from the clay fractions were measured by X-Ray Fluorescence (XRF) using the PANAlytical equipment. About 100 mg of pre-treated sediments were heated at 600 °C to obtain the loss on ignition (LOI), and then were dissolved using a mixture of HNO_3_ + HF on a hot plate. The eluted samples were diluted with 2% HNO_3_ for the major-element measurement. Replicate analyses of selected samples gave a precision of ±2% (2σ) for bulk particles.

### Scanning electron microscope (SEM) analysis

Morphology, elements and composition of clay-fractions (<2 μm) were determined by a LEO1450VP scanning electron microscope (SEM) and an INCA ENERGY 300 X-ray energy spectrometer (EDX). SEM and EDX studies were carried out in the laboratories of the Institute of Geology and Geophysics, CAS.

### Grainsize analysis

All 337 samples were air dried in laboratory. The distribution of grain size was measured for all samples using a Malvern Mastersizer-2000 laser particle analyzer. In this work, grain sizes <2 μm were taken to represent the proportion of clay.

## Additional Information

**How to cite this article**: Zhang, C. *et al.* Clay mineralogy indicates a mildly warm and humid living environment for the Miocene hominoid from the Zhaotong Basin, Yunnan, China. *Sci. Rep.* **6**, 20012; doi: 10.1038/srep20012 (2016).

## Supplementary Material

Supplementary Information

## Figures and Tables

**Table 1:** Summary of all hominoid fossil sites in Yunnan Province, China, and Thailand.

Fossil site	Species	Age (Ma)	Sedimentary unit	Hominoid living environment	References
Zhaotong Basin	*L. cf. lufengensis*	~6.2	Peaty clay below a lignite layer.	Transitional area of mixed forest and grassland with a warm and humid climate.	5,13,19
Lufeng Basin	*L. lufengensis*	6.9−6.2	Alternating thick layers of lignite and peaty clay, silt and sand.	A moist southern subtropical to tropical environment where the climate changed from warm and humid to warm, but drier and then to cooler and more humid.	12,17,18
Yuanmou Basin	*L. hudienensis*	~9.0−7.1	Thick layers of clay, silt and gravel.	A warm and wet predominantly forested habitat with marginal environments consisting of bush-grassland; subtropical, hilly, with some dense forestry and partial transitional bushes and seasonal climate.	9, 10, 11,15,16
Xiaolongtan Basin	*L. keiyuanensis*	12.5–~11.6	Thick layer of lignite.	A warm and wet forested habitat, tropical and subtropical semi-humid evergreen broad-leaved forest.	7,8,14
Chiang Muan basin (Thailand)	*K.cf. chiangmuanensis*	13.5−10	Lignite layers.	An environment with oscillations between tropical woodlands and grasslands.	34,59,60
