# Supplementary material for: Clay mineralogy indicates a mildly warm and humid living environment for the Miocene hominoid from the Zhaotong Basin, Yunnan, China
Source: Sci Rep. 2016 Feb 1;6:20012. doi: 10.1038/srep20012 (PMC4734328; doi:10.1038/srep20012)
Supplement: Supplementary Information [file srep20012-s1.pdf]

Clay mineralogy indicates a mildly warm and humid living environment for the Miocene hominoid from the Zhaotong Basin, Yunnan, China

Chunxia Zhang*[1,2], Zhengtang Guo[1,2], Chenglong Deng[3], Xueping Ji[4,5],Haibin Wu[1], Greig A. Paterson[6], Lin Chang[1], Qin Li[1], Bailing Wu[3], Rixiang Zhu[3]

1. *Key Laboratory of Cenozoic Geology and Environment, Institute of Geology and Geophysics, Chinese Academy of Sciences, Beijing 100029, China*
2. *CAS Center for Excellence in Tibetan Plateau Earth Sciences*
3. *State Key Laboratory of Lithospheric Evolution, Institute of Geology and Geophysics, Chinese Academy of Sciences, Beijing 100029, China*
4. *Department of Paleoanthropology, Yunnan Institute of Cultural Relics and Archeology, Kunming 650118, China*
5. *Institute of Vertebrate Paleontology and Paleoanthropology, Chinese Academy of Sciences, Beijing 100044, China*
6. *Key Laboratory of Earth and Planetary Physics, Institute of Geology and Geophysics, Chinese Academy of Sciences, Beijing 100029, China*

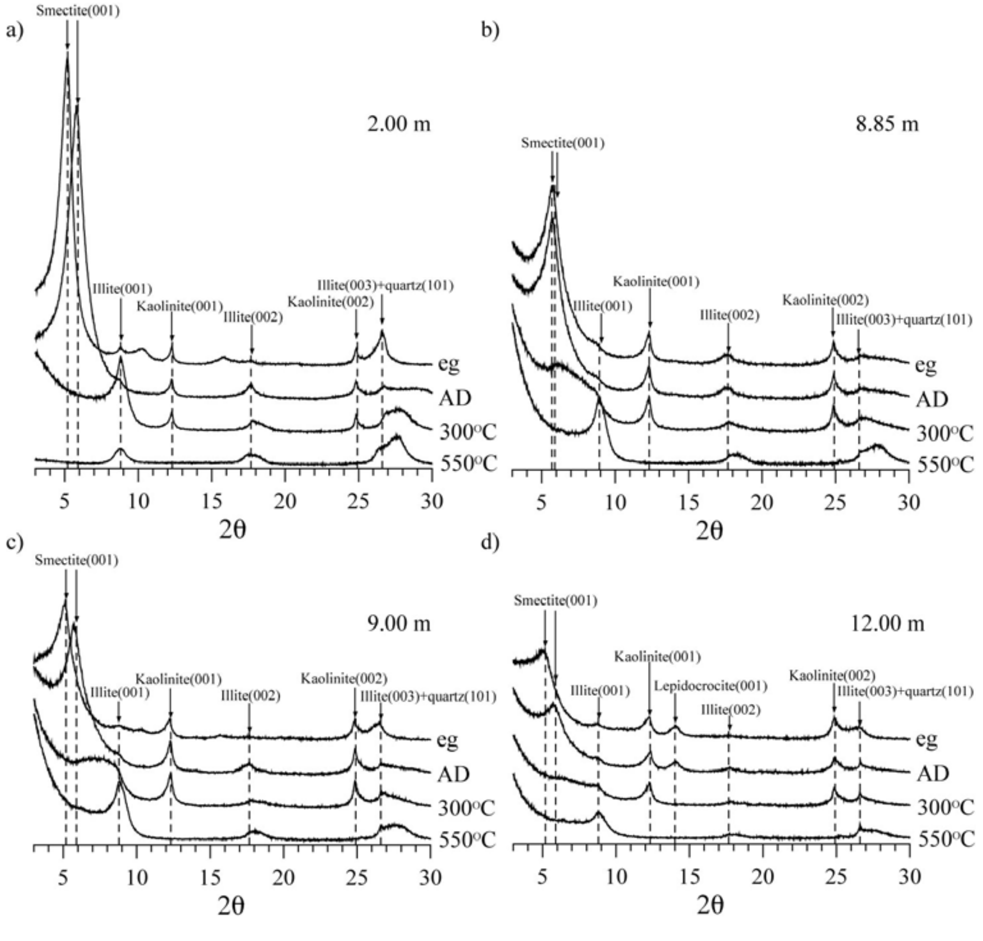

Supplementary Figure S1. Multiple X-ray diffractograms of typical clay-sized

fractions with identification and interpretation of smectite, kaolinite, illite and some quartz from different depths in the STB section.

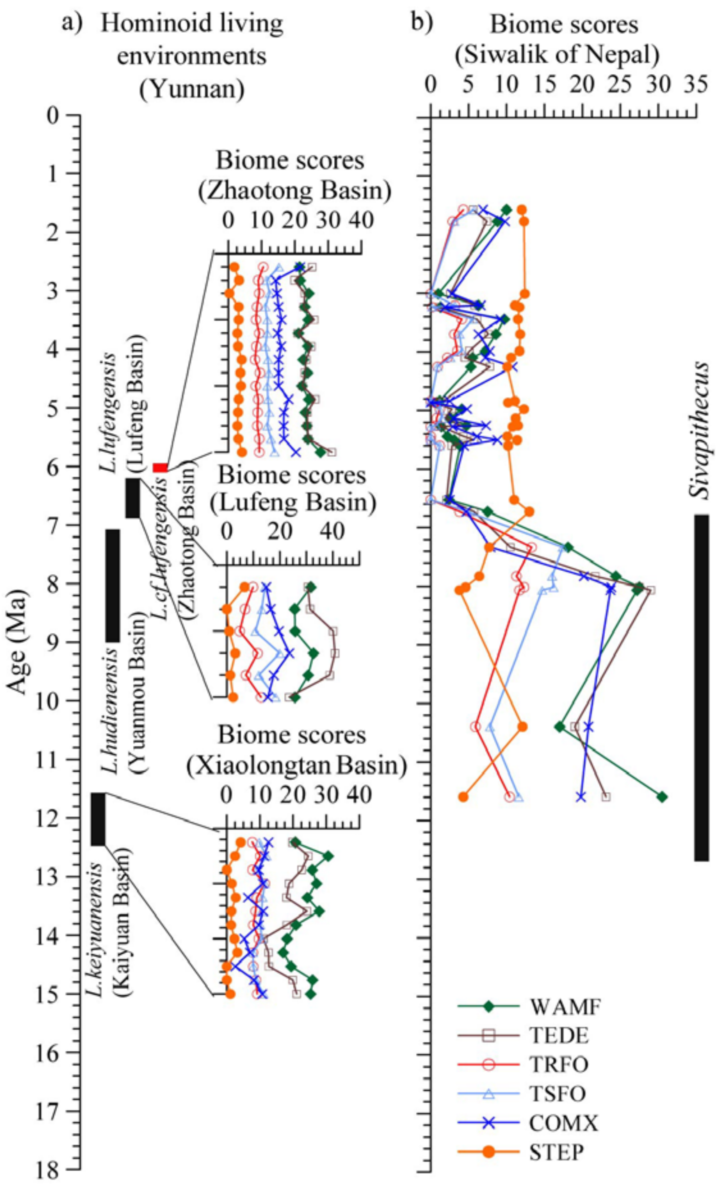

Supplementary Figure S2. Biome vegetation changes in (a) Yunnan Province and (b) the Siwalik Group from Nepal. The Yunnan biomes are reconstructed from the pollen samples of Xiaolongtan [14], Lufeng [17] and Zhaotong [19] Basins, respectively. The biome simulations and results from the Siwalik Group are described in detail by Wu et al., (2014)[38]. Biome types: WAMF, broadleaved evergreen/warm mixed forest; TEDE, temperate deciduous forest; TRFO, tropical rain forest; TSFO, tropical seasonal forest; COMX, cool mixed forest; STEP: steppe.

Supplementary Table S1. Major element composition of clay fractions (< 2μm) from the Zhaotong Basin. Values are percentages.

**Sample ID**	**Depth (m)**	**SiO_2**	**Al_2O_3**	**Fe_2O_3**	**CaO**	**MgO**	**K_2O**	**Na_2O**	**P_2O_5**	**TiO_2**	**MnO**	**LOI**	**Total**
ZT-0	0.00	48.01	23.88	10.95	0.76	1.33	1.73	0.11	0.16	2.17	0.08	10.18	99.54
ZT-80	0.80	48.52	20.87	13.57	0.93	1.36	1.80	0.10	0.45	2.70	0.15	13.36	104.09
ZT-200	2.00	53.96	19.68	11.23	1.76	1.63	1.84	0.11	0.13	1.92	0.03	9.23	101.69
ZT-300	3.00	48.71	18.79	9.13	5.30	1.55	1.69	0.10	0.19	2.01	0.02	11.79	99.44
ZT-450	4.50	51.50	16.90	14.30	1.98	1.4	1.66	0.12	0.22	1.75	0.02	10.32	100.33
ZT-610	6.10	48.98	19.29	10.07	2.47	1.6	1.57	0.11	0.15	1.89	0.02	13.25	99.58
ZT-780	7.80	49.91	21.66	5.06	0.55	1.45	1.68	0.16	0.09	1.84	0.01	15.97	98.53
ZT-810	8.10	49.97	21.94	5.22	0.61	1.47	1.77	0.14	0.09	1.81	0.01	14.85	98.02
ZT-850	8.50	44.86	19.85	4.25	0.56	1.22	1.48	0.14	0.08	1.84	0.01	24.12	98.53
ZT-870	8.70	50.52	20.53	5.60	0.64	1.4	1.56	0.15	0.11	2.32	0.02	16.01	99.00
ZT-875	8.75	47.96	21.04	8.15	1.64	1.65	1.61	0.1	0.14	2.61	0.04	15.71	100.81
ZT-880	8.80	43.16	18.89	14.09	1.88	1.49	1.47	0.11	0.20	2.25	0.14	15.36	99.17
ZT-885	8.85	47.08	21.00	9.27	1.41	1.58	1.71	0.12	0.17	2.15	0.04	13.81	98.49
ZT-890	8.90	49.35	22.24	7.14	1.28	1.70	1.81	0.12	0.13	2.19	0.03	9.59	95.73
ZT-895	8.95	47.58	22.48	7.48	1.29	1.52	1.73	0.14	0.16	1.97	0.03	13.71	98.24
ZT-900	9.00	47.49	21.83	8.29	1.18	1.45	1.71	0.13	0.15	2.00	0.03	13.66	98.10
ZT-906	9.06	47.22	22.45	7.15	1.61	1.48	1.71	0.14	0.15	1.96	0.02	14.00	98.07
ZT-910	9.10	47.42	22.46	7.61	1.53	1.45	1.69	0.13	0.16	2.01	0.02	14.34	98.98
ZT-915	9.15	46.34	21.77	8.27	1.93	1.45	1.66	0.17	0.16	2.03	0.03	14.99	98.94
ZT-920	9.20	53.40	23.34	5.03	0.64	1.43	1.82	0.19	0.13	1.99	0.01	15.57	103.72
ZT-925	9.25	47.59	22.62	6.54	1.38	1.44	1.72	0.13	0.15	1.87	0.02	15.03	98.65
ZT-980	9.80	51.02	22.95	6.07	0.66	1.48	1.50	0.16	0.14	2.50	0.02	12.18	98.85
ZT-1080	10.80	45.24	22.43	11.03	1.30	1.45	1.49	0.14	0.19	2.26	0.06	13.77	99.52
ZT-1140	11.40	45.43	22.31	10.60	1.26	1.30	1.44	0.14	0.21	2.77	0.05	12.75	98.50
ZT-1300	13.00	45.30	22.41	4.40	0.60	1.30	1.53	0.13	0.08	2.15	0.01	20.65	98.70
ZT-1400	14.00	41.37	19.19	1.73	1.01	0.81	1.51	0.15	0.03	1.29	0.00	32.09	99.33
